# The Effect of Religiosity on Life Satisfaction in a Secularized Context: Assessing the Relevance of Believing and Belonging

**DOI:** 10.1007/s13644-016-0282-1

**Published:** 2017-01-17

**Authors:** Josje ten Kate, Willem de Koster, Jeroen van der Waal

**Affiliations:** Department of Public Administration and Sociology, Erasmus University Rotterdam, P.O. Box 1738, 3000 DR Rotterdam, The Netherlands

**Keywords:** Believing, Belonging, Life satisfaction, Religion, Religious affiliation, Subjective well-being

## Abstract

The positive relationship between religiosity and life satisfaction is well-established. This relationship is, however, likely to vary across cultural contexts and different religious affiliations. Furthermore, research is needed to uncover *why* religion is relevant for life satisfaction. Addressing these issues, we investigate what dimensions of being religious play a role in the life satisfaction of individuals with different religious affiliations, including the understudied Muslim category, in the highly secularized Dutch context. We examine ‘believing’, which captures how religion provides meaning and a coherent worldview, and ‘belonging’, which comprises both cultural benefits of being embedded in a congregation with a shared framework of meaning and structural benefits due to more social ties. Analyses of the NEtherlands Longitudinal Lifecourse Study (*n* = 5312) first indicate that Muslims display significantly lower life satisfaction than the non-religious, which appears to be due to their underprivileged social position rather than intra-religious factors of believing and belonging. Second, we find that Catholics experience significant life satisfaction benefits compared to those who are not religious, and that only belonging plays a role in this association. Next to the beneficial effect of the structural aspect of belonging, which revolves around social ties, a cultural aspect of religious belonging appears to be salient, suggesting that an important life satisfaction advantage of religious communities lies in their ability to foster a sense of solidarity and commitment through a shared framework of meaning. We make several recommendations for further research based on these findings.

## Introduction

Many studies have found that religion has beneficial effects on various facets of mental well-being (Ellison and Henderson 2011; Witter et al. 1985; Yeary et al. 2012). Research has, for instance, shown that depression (McCullough and Smith 2003), perceived quality of life (Ferriss 2002), and life satisfaction (Lim and Putnam 2010; Willits and Crider 1988) are positively related to measures of religiosity. Although the claim that religion has a positive effect on life satisfaction thus finds widespread support in the academic literature, the relationship appears to be in need of further scrutiny.

Although most studies into the association between religion and life satisfaction have been conducted in the US, this relationship appears to depend on the context. Okulicz-Kozaryn (2009), for instance, found that the association is weaker in countries that are less religious. Similarly, it is possible that the degree to which a country is pluralistic plays a role in determining the effect of religion on life satisfaction. It is for instance found that in secularized, pluralistic contexts, where a variety of lifestyles are accepted and personal freedom is encouraged, traditional religions meet fierce criticism (Achterberg et al. 2009; Houtman et al. 2011; Inglehart 2000; Ribberink et al. 2013). This would lead to the expectation that being religious does not contribute to life satisfaction in such a context. In contrast, it is also conceivable that being religious offers a sense of security and other resources that are much-needed in such a highly pluralistic and secularized context, and that religion is therefore an especially important *benefit* to life satisfaction. Taking these contrasting argumentations into account, it is thus not yet clear whether being religious affects life satisfaction to the same extent in a country that is both secularized and pluralistic as in the well-studied American context and, if it does, whether it increases or decreases life satisfaction.

A second issue that deserves attention concerns the fact that significant variations might exist across different religions (Cohen 2002). For Muslims, who are under-represented in this field of research (Abdel-Khalek 2010), it can on the one hand be argued that their religion exposes them to considerable stigmatization and negative treatment in contemporary Western societies (Razack 2008; Spruyt and Elchardus 2012; Van Bohemen et al. 2012). On the other hand, their religion may also prove to be a great comfort in a hostile environment, as such contributing to their satisfaction with life. As research has primarily focused on Christianity (Koenig et al. 2001), the life-satisfaction effect of being Islamic is understudied.

Finally, and perhaps most importantly, several authors stress the need for research that helps us to understand *why* religion influences mental well-being measures like life satisfaction (George et al. 2002). Ellison (1991), for instance, claims that “researchers remain far from a consensus on which specific dimensions of religiosity contribute to psychological well-being and subjective perceptions of life quality” (p. 80). Similarly, Lim and Putnam (2010) confirm that “much theoretical and empirical controversy surrounds the question of how religion actually shapes individuals’ well-being” (p. 914). As a consequence, there is a pressing need for research that delves deeper into why religion influences life satisfaction and advances our insight into which characteristics of the religious are actually relevant for understanding how satisfaction with life is shaped.

Given the issues discussed above, three interrelated questions arise: (1) does religion enhance or decrease life satisfaction in a highly secularized and pluralistic context; (2) do variations of this life-satisfaction effect exist across different religious affiliations; and, most importantly, (3) what dimensions of religion play a role in explaining the relationship between religion and life satisfaction? We answer these questions by analyzing representative Dutch survey data that not only allow Catholics and Protestants to be compared with the non-religious, but also includes the understudied category of Muslims.

In line with previous studies (see, e.g., Achterberg et al. 2009; de Koster et al. 2010; Houtman and Mascini 2002), we use the Netherlands as a strategic case to study religion in a secularized context. The Netherlands is one of the most secularized countries in the world (Inglehart and Baker 2000), which is not only illustrated by its low number of religious individuals (Lechner 1996; Norris and Inglehart 2004), but also by the fact that traditional Christian values are fiercely criticized and the identity of religious individuals is frequently berated in the public domain (Houtman et al. 2011; Ribberink et al. 2013). In contrast with the US, where most research is conducted, the religious do not hold a privileged social position in the Netherlands, which could have important implications for the impact of religion on life satisfaction (Cragun et al. 2016).

Dutch society is not only exceptionally secularized, but also pluralistic. Next to being well-known for its openness to a wide variety of lifestyles and identities (Houtman et al. 2011), it is characterized by religious diversity: the minority of people in the Netherlands that is religious does not consist of one homogeneous group but is made up of individuals with different religious affiliations (Chaves and Gorski 2001; Inglehart and Baker 2000). Protestants, Catholics, and, more recently, Muslims, are the largest religious groups in the Netherlands (Rath et al. 1999). Individuals belonging to different religious affiliations are furthermore segregated geographically, with Protestants mainly living in the Dutch ‘Bible Belt’, Catholics in the south of the Netherlands, and Muslims in the big cities (Schmeets and Van Mensvoort 2015).

In order to explain how religion could influence life satisfaction in such a secularized and pluralistic context, we test various hypotheses derived from the relevant literature, which focus on two dimensions of religion: (1) providing a coherent worldview (‘believing’), and (2) integrating individuals into a (religious) community (‘belonging’), discerning between cultural benefits in the form of being embedded in a community of like-minded peers and a structural aspect of belonging which revolves around social ties.

## Believing and Life Satisfaction: Coherent Worldview and Meaning

According to many authors, one of the functions of religious ‘believing’ is that it provides a coherent interpretation and meaning in one’s life (e.g. Ellison 1991). This coherent worldview is not only considered to contribute to feelings of well-being in general, but may be especially beneficial in a pluralistic context such as the Netherlands. In this section, we will elaborate on two components of religion that contribute to offering such a meaning system to individuals: religious beliefs and private religious practices.

### Religious Beliefs

One characteristic of religious individuals is that they adhere to religious beliefs that offer interpretations of the empirical world and the ‘right’ role of the individual in it (Ellison and Levin 1998). Indicating that such a belief system may contribute to explaining why religiosity might be positively related to life satisfaction, believers have reported higher levels of happiness than non-believers in a variety of studies (see, e.g., Bradshaw and Ellison 2010; Ellison et al. 2001; Murphy et al. 2000).

An explanation for this can be found in religiosity’s potential to cope with, and thus alleviate, feelings of uncertainty. Extending the ‘insecurity theory’ proposed by Norris and Inglehart (2004), Immerzeel and Van Tubergen (2013) state that religiosity may help to appease an increased experience of insecurity. Both individual and contextual conditions affect such experiences, and are related to an increased need for guidance and comfort (Immerzeel and Van Tubergen 2013). Pluralization contributes to the erosion of a monolithic system of beliefs and meaning (Berger 1967), and can be seen as an example of a contextual source of insecurity. Some authors even argue that secularized, pluralistic societies bring “a growing concern for the meaning and purpose of life” (Inglehart 2000, p. 224), bringing about a pressing sense of instability and insecurity. This means that, in contemporary pluralistic societies, individuals may experience a lack of guidance and are less confident that problems will be overcome.

Following both classic and modern sociologists (Ellison 1991; Weber [1922] 1963; Zijderveld 2000), it can be expected that religious individuals are less affected by this, since religious beliefs help make events more comprehensible and bestow meaning on seemingly mundane affairs. Hence, religious individuals are less likely to see the problems they encounter as a threat. As such, religious beliefs can be important coping devices, allowing people to adjust to major life events more easily and offering a more stable view of the world, so reducing levels of stress (Ellison 1991; George et al. 2002). In addition, an individual’s life is bestowed with special value (Ellison 1991), because she adheres to moral guidelines provided by her religious faith. This adds to the idea that one is a good, virtuous person (Solomon et al. 1991), and may enhance an individual’s sense of self-worth (Crocker et al. 2003).

Further strengthening the function of offering a sense of security, traditional religions such as Christianity are based on the idea that a divine force or God will ensure that ‘all goes well’ or at least will be well in the future, thus providing a sense of safety (Bradshaw et al. 2010). Feelings of stress about the future are in this way further reduced, contributing to overall well-being (Ellison et al. 2011). Believing in God may also be linked to self-worth through the conviction that one is loved and valued by Him (Crocker et al. 2003). A positive relationship between God images and self-esteem has been reported (Benson and Spilka 1973; Francis et al. 2001), suggesting that religious beliefs in a God contribute to life satisfaction.

Based on the above we expect religiously affiliated individuals in the Netherlands to display higher levels of life satisfaction than those who are not religious because of their religious beliefs (*hypothesis 1a*).

### Private Religious Practices

Along with religious beliefs, practicing religion in a private setting is a dimension of religiosity that can play a role in enhancing a sense of security and coherence. According to Ellison and Levin (1998), religious activities like praying are central to developing a relationship with a divine other. According to attachment theory, religious attachment figures like God may serve as a source of support and companionship in stressful times (Pargament et al. 1988) and provide individuals with feelings of love and safety (Bradshaw et al. 2010). When people are thus in need of guidance, they can rely upon their relationship with God, which encourages feelings of hope, a sense of control (Bradshaw and Ellison 2010), and an overall feeling of tranquility (Ellison et al. 2014). Through prayer and divine interaction, individuals may further gain a heightened sense of self-worth and control, as it strengthens the feeling that they are valued and helped by a divine force (Ellison 1991). This is empirically supported by Maltby et al. (1999), who found that personal prayer plays an important role in accounting for variations in depressive symptoms, anxiety, and self-esteem.

In addition to praying, reading religious texts may contribute to explaining why religiosity is associated with higher levels of life satisfaction; through reading such texts, individuals may feel connected to the characters in them, especially if they face similar issues (Ellison 1991). In this way, identifying with biblical figures may help in dealing with a variety of problems: through “religious role-taking” (Bradshaw and Ellison 2010, p. 198) individuals may get ideas about how to behave and think in order to solve their problems. By interpreting their own issues in terms of the situation of a biblical figure, individuals may also feel ‘less alone’ in facing them (Ellison and Levin 1998). The moral codes and guidelines for behavior provided by religious texts may further help to solve such issues, as well as help individuals avoid ‘risky’ situations and behaviors in the first place (Ellison and Levin 1998). Reading religious texts is thus not only useful for avoiding and resolving actual problems, but may also lead to enhanced perceptions of control by inducing the faith that one has the ‘tools’ to also do so in the future.

Following this argument, we expect that praying and reading religious texts such as the Bible or Koran would explain the beneficial effect of religious affiliation on life satisfaction (*hypotheses 1b* and *1c,* respectively).

## Belonging and Life Satisfaction: Cultural and Structural Factors

In addition to the meaning and guidance that religious beliefs and practices offer, being religiously affiliated has an important integrative function (Durkheim [1915] 1965). In order to illustrate how this might explain the relationship between religiosity and life satisfaction, we will discuss two types of benefit of belonging to a religious congregation: cultural and structural.

### The Cultural Aspect of Belonging: Embeddedness in a Community of Like-Minded Peers

A first, cultural benefit of being a member of a religious congregation lies in the binding power of a common framework of meaning and the sense of belonging that comes with it. Religious narratives, rites, and rituals form a system of symbols, which is central to creating and maintaining a sense of “togetherness” (McMillan and Chavis 1986: 12). Symbols or social conventions like these strengthen a sense of group membership and foster identification with the group, or, as Krause and Wulff (2005) phrase it: “group prayer, sermons, hymns (…) continuously remind church members they are part of a larger family that is bound together by a common faith and sense of commitment to each other” (pp. 80–81). Religiously affiliated individuals can thus be said to be embedded in a community of like-minded peers or a ‘family’ with whom they share key norms and values, which is fostered by their participation in their congregation and religious services.

According to extant literature on communities, belonging to a community where the members share the same worldview may have several advantages when it comes to life satisfaction. First, being part of a congregation with a common framework of meaning may incite a sense of intimacy. Group members feel that they matter to each other, fit within the group, and are accepted by its members, thereby fostering a sense of ‘closeness’ (McMillan and Chavis 1986). Perceptions of being accepted by, and important to, like-minded peers are positively associated with emotional well-being (Glover et al. 1998) and general self-worth (Bagwell et al. 1998). This perception of intimacy is also protected by group boundaries, which safeguard the group’s beliefs and the safety of its members (McMillan and Chavis 1986). This reflects another advantage of belonging to a group of like-minded peers: a feeling of emotional security. Belonging to a community is associated with a place of warmth and comfort (Bauman 2001), making members feel ‘at home’. In addition, group-members are expected to support and stand up for each other (McMillan 1996), which adds to the feeling that one can depend on fellow community-members and is accepted by them. This creates a safe environment in which self-disclosure is encouraged (de Koster 2010; McMillan 1996), and this has been found to be conducive to good mental health (Larson et al. 2015). Based on these advantages to general mental well-being, we expect that religiously affiliated individuals are more satisfied with their lives because they participate in a religious congregation with a shared culture (*hypothesis 2*).

### The Structural Aspect of Belonging: Social Ties, Support, and (Less) Loneliness

Along with the benefits of being embedded in a community with a shared culture, there is a structural advantage to participating in a religious congregation, which lies in the larger amount of social relations that churchgoers obtain: belonging to a religious community may result in social ‘spill-overs’ in other (that is, non-religious) domains (Putnam 1995; Putnam and Campbell 2010; Yeary et al. 2012). As Schwadel and Falci (2012) argue: “an additional social resource is the ability of churches to help congregants make new connections across diverse social networks” (p. 22). For example, the religious are more embedded in a cohesive neighborhood (McCullough and Smith 2003). By stimulating embeddedness in a cohesive neighborhood, belonging to a religious community serves as a potential source of social benefits in terms of self-esteem and social support (Thoits 2011), as such enhancing general well-being (Ross and Jang 2000). Accordingly, we expect religiously affiliated individuals to report higher levels of life satisfaction because they are more embedded in a cohesive neighborhood (*hypothesis 3a*).

In addition to being more embedded in a cohesive neighborhood, religious individuals may have more family ties than non-religious individuals. According to Mahoney et al. (2003), religion is associated with the “sanctification of family relationships” (p. 221), since it promotes attributing a special meaning to marriage and parenthood. Because of this, religious individuals may have more social ties in the family domain. The sharing and reaffirmation of such norms and practices within the religious community are also a reason why religious belonging is related to having more family ties (Waite and Lehrer 2003), which, like embeddedness in a cohesive neighborhood, may lead to higher self-esteem (Demo et al. 1987), more social support, less loneliness, and a greater sense of control (Ross et al. 1990). Accordingly, we hypothesize that religiously affiliated individuals report higher levels of satisfaction with their lives because they have more social ties in the family domain (*hypothesis 3b*).

Social support is not only provided through family ties and neighborhood embeddedness stimulated by belonging to a religious congregation, it is also offered by the religious community itself (Krause and Wulff 2005; Nguyen et al. 2016). Belonging to such a community offers a variety of tools that are helpful in solving problems and play a noteworthy role in coping strategies employed in times of stress (Kvande et al. 2015; Nguyen et al. 2013). Through the provision of social support in the form of love, caring, and sympathy, community membership is associated with higher levels of self-esteem and a sense of control (Berkman 1995), leading to a positive influence on overall well-being. This suggests the religious generally have a greater availability of social resources that aid coping with encountered problems and alleviate feelings of loneliness (Lynch et al. 2000; Thoits 2011). This leads us to expect that individuals belonging to a religious community will report higher levels of life satisfaction because they have a greater availability of social support (*hypothesis 3c*) and experience less loneliness (*hypothesis 3d*), in addition to the potential spill-over effects of being embedded in a religious congregation hypothesized above.

## Data and Measures

We have used the first wave of the NEtherlands Longitudinal Lifecourse Study (NELLS). The response rate was 56%, which is average for this type of survey in the Netherlands (De Graaf et al. 2010a). The data were collected in 2009 from 5312 individuals from 35 municipalities in the Netherlands that were selected in order to cover different regions and varying degrees of urbanization (including the four biggest cities in the Netherlands). The participants (aged 14–49 years; the sample is relatively young because this is the first wave of an envisaged panel study) were randomly selected using the population register (De Graaf et al. 2010b). In this process, respondents of Moroccan and Turkish origin were purposely oversampled. Next to 2556 native Dutch individuals, the dataset contains non-native respondents of Moroccan (1164), Turkish (1137), other Non-Western (226), and Western (229) origin. The sample is especially useful for our analyses, because it allows us to include the typically under-represented religious group of Muslims, enabling the examination of a broader scope of religious affiliations than is common in the relevant literature. To re-weight the different ethnic groups to the national redistribution (De Graaf et al. 2010a), we applied the weight provided with the data (w1cweight2). We used multiple imputation to account for missingness in our data, which we will discuss in more detail in the results section.

Our dependent variable, life satisfaction, was measured with a commonly-used scale (Diener 1994; Veenhoven 1996). Respondents were asked to indicate to what extent (ranging from 1 = ‘Completely agree’ to 5 = ‘Completely disagree’) they agreed with the following statements: ‘In most respects, my life is ideal’; ‘My living conditions are excellent’; ‘Taking all things together, I am satisfied with my life’; and ‘I have achieved the most important things I expected from life so far’. A factor analysis produced a first factor with an eigenvalue of 2.79 explaining 70% of the variance. For respondents without missing values on any of the four items, a scale was constructed with the reverse-coded items, so that higher scores represented higher levels of life satisfaction (Cronbach’s alpha = 0.86).

In order to disentangle how religious affiliation is related to life satisfaction, we made a distinction between the different *religious affiliations* that are represented in the dataset. Respondents were categorized as ‘Catholic’, ‘Protestant’, ‘Islamic’, ‘Other’ (including Judaism, Hinduism, and Buddhism), or ‘No religious affiliation’ (reference category).

As believing in a God or higher power is generally indicated to be a dimension of religiosity that is related to health outcomes (see, e.g., George et al. 2002, p. 198), this is what we focused on when constructing our measure of *religious beliefs.* An item was used that asked the respondents to indicate which of the statements came the closest to their own conviction: ‘There is a God’; ‘There is a kind of life spirit or force’; ‘I don’t know what to think’; or ‘I do not think there is a spirit or God or life force’. A categorical variable was then created, with the last answer being the reference category.

Following Levin (1999) and Maselko and Kubzansky (2006), private religious practices were measured by examining religious activities undertaken outside public religious institutions and often performed at home. The NELLS contains two indicators of such private religious activities: *praying* and *reading the Bible or Koran*. For *praying*, the respondents were asked whether they had prayed in the last three months (0 = ‘No’; 1 = ‘Yes’). Similarly, they were asked whether they had *read the Bible or Koran* in the past three months, which also resulted in a dichotomous variable (0 = ‘No’; 1 = ‘Yes’).

Since religious attendance can be taken as a proxy of how well individuals are integrated into a religious community (Brisette et al. 2000: 59–61), and as the advantages of being embedded in a community with a shared framework of meaning are thus more likely to occur among those who attend church more often (Ellison and George 1994), we used *religious attendance* to measure the extent to which the respondents belonged to a religious community of like-minded peers. Net of effects of social support, loneliness, and embeddedness in the neighborhood and family, we use attendance at religious services as a measure of the cultural aspect of belonging in this study. The respondents indicated on a seven-point scale how frequently they attended religious services in a church, synagogue or mosque, with possible responses ranging from ‘never’ (1) to ‘multiple times per week’ (7).

To include *embeddedness in a cohesive neighborhood*, we used four items that measured to what extent (ranging from 1 = ‘Completely true’ to 4 = ‘Not true at all’) the respondents felt that the following statements were true for their neighborhood: ‘People in this neighborhood greet each other’; ‘You can trust the people in this neighborhood’; ‘In general, the people in the neighborhood get along well’; ‘A lot of people know each other in this neighborhood’; ‘The people here want to help each other’; and ‘The people in this neighborhood would say something if youths caused any inconvenience’. A factor analysis produced a first factor with an eigenvalue of 3.57, explaining 59% of the variance. The scores were reverse-coded so that higher scores on the scale (Cronbach’s alpha = 0.85) represented higher levels of *embeddedness in a cohesive neighborhood*.

Two aspects of family life were used to measure social ties in the family domain: marital status and having children. First, *marital status* was included as a categorical variable (1 = ‘No partner (reference category)’; 2 = ‘Married and cohabiting’; 3 = ‘Unmarried and cohabiting’; and 4 = ‘Not cohabiting (married or unmarried)’). We also used *having children* (0 = ‘No’ and 1 = ‘Yes’) to measure social ties in the family domain.

*Social support* was measured by combining three items, which indicated to what extent (ranging from 1 = ‘Very much’ to 4 = ‘Not at all’) the respondents felt that the following statements applied to their life: ‘There are enough people to whom I feel closely connected’; ‘I have a lot of people who I can trust completely’; and ‘There are enough people who I can fall back on in bad times’. A factor analysis produced a first factor with an eigenvalue of 2.21, explaining 74% of the variance. Only respondents with scores on all three items were included in the final scale, for which the scores were reverse-coded so that higher scores represented higher levels of social support (Cronbach’s alpha = 0.81).

*Loneliness* was included by using the following three items: ‘I experience a void around me’; ‘I miss people around me’; and ‘I often feel abandoned’. The respondents were asked to indicate to what extent these statements applied to their life, with answers ranging from ‘Very much’ (1) to ‘Not at all’ (4). The factor analysis produced a first factor with an eigenvalue of 2.20, explaining 73% of the variance. A scale (Cronbach’s alpha = 0.80) was created for the respondents with scores on all three items. The scores were reverse-coded so that higher scores indicated higher levels of *loneliness*.

In order to control for the influence of education (see, e.g., Hartog and Oosterbeek 1998), we included in the analyses the number of years formally required to complete the highest attained level of education. Gender (0 = ‘Male’ and 1 = ‘Female’) and age in years were also included as controls (for the influence of gender on happiness, see Stevenson and Wolfers 2008; for age, see Fugl-Meyer et al. 2002). To take a potential curvilinear effect of age into account (see, e.g., Blanchflower and Oswald 2008), we included a centered measure of age and a quadratic term of this measure in our analyses. Additionally, household income (see, e.g., Howell and Howell 2008) in 16 categories (ranging from 1 = ‘Less than 150 Euros per month’ to 16 = ‘7000 Euros or more per month’) and ethnicity (see, e.g., Barger et al. 2008), coded as native Dutch, Moroccan, Turkish, non-Western other, and Western other, were controlled for in our analyses.

## Results

Table 1 provides descriptive statistics for the variables used in our analyses. Because some variables contained several missing cases, we used multiple imputation to fill in these values.

**Table 1:** Descriptive statistics before multiple imputation

	Mean	SD	Range	*n*
Life satisfaction	3.82	0.66	1–5	4885
No religious affiliation (ref.)	0.61	0.49	0–1	5310
Catholic	0.12	0.32	0–1	5310
Protestant	0.14	0.35	0–1	5310
Islam	0.09	0.28	0–1	5310
Other	0.05	0.21	0–1	5310
No beliefs (ref.)	0.19	0.39	0–1	4860
Belief in God	0.32	0.47	0–1	4860
Belief in life spirit/force	0.25	0.43	0–1	4860
Don’t know what to believe	0.24	0.43	0–1	4860
Praying	0.39	0.49	0–1	4860
Reading Bible/Koran	0.24	0.42	0–1	4863
Attendance at religious services	2.44	1.86	1–7	5311
Social support	3.18	0.56	1–4	4843
Loneliness	1.80	0.62	1–4	4840
No partner (ref.)	0.32	0.47	0–1	5310
Married cohabitation	0.34	0.47	0–1	5310
Unmarried cohabitation	0.19	0.40	0–1	5310
No cohabitation	0.14	0.35	0–1	5310
Children	0.45	0.50	0–1	4760
Neighborhood social ties	3.31	0.59	1–4	5312
Education	13.43	4.00	0–22	5311
Age	29.87	8.96	14–49	5312
Gender (female)	0.49	0.50	0–1	5312
Household income	6.25	3.27	1–16	4714
Native Dutch (ref.)	0.79	0.41	0–1	5312
Moroccan	0.03	0.16	0–1	5312
Turkish	0.03	0.17	0–1	5312
Non-Western other	0.08	0.27	0–1	5312
Western other	0.07	0.26	0–1	5312

A total of 10 imputed datasets were created, based on an imputation model including the variables listed in Table 1 and other potentially relevant items without missing values.^1^ Because our dependent variable, life satisfaction, also contained missing values and its outcome was predicted well by the variables included in our imputation model, life satisfaction was included as an imputed variable as well. Note that our results are highly robust, evidenced by four alternative analyses: (1) analyses based on an imputation model that also included the additional items next to those in our regression analyses, but without imputation of life satisfaction; (2) a model that did not make use of additional variables next to those in the final regression analyses, with imputation of life satisfaction; (3) a model without additional items without imputation of life satisfaction; (4) and, finally, we handled missing data using listwise deletion instead of multiple imputation. All four alternative analyses corroborate and reject the same hypotheses as our analyses presented in Table 2.

**Table 2:** Explaining the relationship between religion and life satisfaction (OLS regression analyses; *n* = 5312; unstandardized coefficients shown; standard errors in parentheses)

	Model 1	Model 2	Model 3	Model 4	Model 5	Model 6
*Independents*						
Constant	3.83***	3.00***	3.00***	2.95***	2.52***	2.60***
	(0.02)	(0.07)	(0.07)	(0.08)	(0.11)	(0.14)
No religious affiliation (ref.)						
Catholic	0.08*	0.10**	0.13***	0.09*	0.08*	0.04
	(0.04)	(0.03)	(0.04)	(0.04)	(0.04)	(0.03)
Protestant	0.03	0.03	0.08	0.00	−0.01	−0.03
	(0.04)	(0.04)	(0.06)	(0.06)	(0.05)	(0.05)
Islam	−0.20***	−0.06	−0.02	−0.04	−0.08	0.01
	(0.04)	(0.07)	(0.08)	(0.08)	(0.08)	(0.08)
Other	−0.10	−0.02	0.04	−0.04	−0.06	−0.06
	(0.06)	(0.06)	(0.07)	(0.08)	(0.07)	(0.07)
No beliefs (ref.)						
Belief in God			0.01	−0.01	−0.03	−0.02
			(0.05)	(0.05)	(0.05)	(0.05)
Belief in life spirit/force			0.01	0.00	−0.01	−0.01
			(0.04)	(0.04)	(0.04)	(0.03)
Don’t know what to believe			0.03	0.03	0.00	0.02
			(0.04)	(0.04)	(0.04)	(0.03)
*Praying*			−0.08*	−0.10**	−0.10*	−0.04
			(0.04)	(0.04)	(0.04)	(0.03)
Reading Bible/Koran			0.00	−0.07	−0.05	−0.05
			(0.04)	(0.04)	(0.04)	(0.04)
Attendance at religious services				0.05***	0.04***	0.03**
				(0.01)	(0.01)	(0.01)
Embeddedness in cohesive neighborhood					0.13***	0.09***
					(0.02)	(0.02)
No partner (ref.)						
Married cohabitation					0.28***	0.22***
					(0.04)	(0.04)
Unmarried cohabitation					0.17**	0.13**
					(0.04)	(0.04)
No cohabitation					0.19***	0.14**
					(0.04)	(0.04)
Children					0.00	0.03
					(0.04)	(0.03)
Social support						0.23***
						(0.03)
Loneliness						−0.27***
						(0.02)
*Controls*						
Education		0.02***	0.02***	0.02***	0.02***	0.01***
		(0.00)	(0.00)	(0.00)	(0.00)	(0.00)
Age		−0.02***	−0.02***	−0.02***	−0.02***	−0.02***
		(0.00)	(0.00)	(0.00)	(0.00)	(0.00)
Age^2^		0.00***	0.00***	0.00***	0.00***	0.00***
		(0.00)	(0.00)	(0.00)	(0.00)	(0.00)
Gender (female)		0.02	0.02	0.03	0.01	0.01
		(0.02)	(0.02)	(0.02)	(0.02)	(0.02)
Household income		0.07***	0.07***	0.07***	0.05***	0.04***
		(0.01)	(0.01)	(0.01)	(0.01)	(0.01)
Native Dutch (ref.)						
Moroccan		0.03	0.04	0.04	0.07	0.02
		(0.07)	(0.07)	(0.07)	(0.07)	(0.07)
Turkish		−0.05	−0.04	−0.05	−0.02	−0.01
		(0.06)	(0.07)	(0.06)	(0.06)	(0.06)
Non-Western other		−0.11*	−0.10	−0.09	−0.04	−0.05
		(0.05)	(0.05)	(0.05)	(0.05)	(0.05)
Western other		−0.05	−0.05	−0.04	0.01	0.04
		(0.05)	(0.05)	(0.05)	(0.05)	(0.05)
Mean R^2^	0.01	0.11	0.11	0.12	0.15	0.28

* *p* < 0.05; ** *p* < 0.01; *** *p* < 0.001 (two-sided tests for significance)

After imputing missing data, multivariate linear regression analyses of pooled results were conducted to determine whether, and why, religiously affiliated individuals experience higher levels of life satisfaction than those who are not religiously affiliated (see Table 2). First, we assess whether religiosity affects life satisfaction at all, and if so, whether this effect varies across the different religious affiliations included in our analyses (see models 1 and 2). In the models that follow (models 3 to 6) we assess which dimensions of religion actually play a role in shaping life satisfaction, discerning between ‘believing’ and ‘belonging’. VIF scores (ranging from 1.09 to 4.34) indicate that multicollinearity is not an issue in any of our analyses.

Starting with the question of whether religion plays a role in shaping individual levels of life satisfaction in the Netherlands, we first observe models 1 and 2, which show the effect of religious affiliation on life satisfaction *without* controlling for the influence of our control variables (Model 1), and *with* their influence taken into account (Model 2). Both Model 1 and 2 show there is no universal effect of religion on life satisfaction in this study. Whereas being Catholic is positively related to life satisfaction, individuals belonging to a Protestant, Islamic or other religious affiliation are not more satisfied with their lives compared to individuals who are not religious. This shows that considerable variations exist in the extent to which different religions offer resources that benefit satisfaction with life, and that differentiation between religions is therefore crucial.

This point is further illustrated by the fact that we find a negative relationship between being Muslim and life satisfaction: Islamic individuals appear to be generally *less* satisfied than those without a religious affiliation. When comparing Model 1 to Model 2, we see that this relationship disappears when we control for the influence of education, age, gender, household income, and ethnicity: the effect of being Muslim falls from −0.20 to −0.06 and is no longer significant. Based on this notable decrease of the effect of being Muslim, we can conclude that indicators of socioeconomic position and ethnic minority status play an important role in explaining the strong, negative effect of Islam in Model 1, as they take over a large part of the initial effect. This suggests that, in the Netherlands, Muslims do not have lower levels of life satisfaction because of intra-religious factors of either believing or belonging, but because of their underprivileged social position.

Focusing on Catholic individuals, who are more satisfied with life than individuals who are not religiously affiliated, we continue by examining whether the relationship identified can be explained by aspects of either ‘believing’ or ‘belonging’ in models 3 to 5. Turning to Model 3, in which indicators of believing are included, it is clear that the association of being Catholic with life satisfaction was not reduced after adding these indicators. In fact, the coefficient in Model 3 (0.13) is larger than in Model 2 (0.10). This means that the higher levels of life satisfaction of Catholic individuals cannot be explained by the indicators of believing added in Model 3. *Hypothesis 1a*, which states that religiously affiliated individuals are more satisfied with their lives because of their religious beliefs, must therefore be rejected. The same applies to reading the Bible or Koran (*hypothesis 1c*): it does not appear to explain why Catholics are generally more satisfied with their lives than those who are not religious.

When it comes to praying, which is central to *hypothesis 1b*, Table 1 shows that only 39% of the respondents report to have prayed in the last three months. Inspecting the relationship between praying and religious affiliation more closely shows that this is mainly because of the large share of individuals who are not religiously affiliated, which clearly speaks to the secularized context of the Netherlands. Praying is common among the religious groups in our sample: 54% of the Catholic respondents, 88% of the Protestants, 80% of the Muslims, and 91% of the respondents who belong to a different religious affiliation prayed in the past three months.

Although prayer is common among the religiously affiliated in this study, Table 2 indicates that we cannot confirm *hypothesis 1b*. In fact, Model 3 shows that those who pray actually report lower levels of life satisfaction, instead of the higher levels hypothesized. These findings suggest that although religion may offer a coherent worldview and meaning through religious beliefs and private religious practices, this function of religion does not provide Catholics with higher levels of life satisfaction in the secularized, pluralistic context of the Netherlands. If anything, the increased coefficient of being Catholic in Model 3 suggests a suppressor-effect plays role here. We will further elaborate on this in the concluding section.

Having examined aspects of ‘believing’, which do not appear to play a role in explaining why Catholics are more satisfied with their lives than the non-religious, we now turn to the aspects of ‘belonging’ for an explanation of this relationship in models 4, 5 and 6. Model 4, in which the influence of belonging to a religious community of like-minded peers is tested, shows the role of attendance at religious services. When attendance is included, the association of being Catholic with life satisfaction decreases from 0.13 to 0.09. This finding shows that religious belonging indeed plays a role in explaining why Catholics are generally more satisfied with their lives than the non-religious. The question that remains at this point concerns the different aspects of religious belonging: how do cultural aspects, focused on being embedded in a community of like-minded peers, and structural aspects, namely social ties, availability of support and alleviation of loneliness, each play a role?

The effect of attendance at religious services that remains after taking structural factors into account can reasonably be understood as a cultural aspect of belonging, as argued in the theoretical section. This means that for testing *hypothesis 2* (on the role of a cultural aspect of belonging), we have to add the structural factors associated with belonging to a religious community to our analyses. Therefore, we first examine the role of social spill-overs in the family and neighborhood and social support and loneliness. When it comes to the neighborhood, exploratory analyses show that religious attendance and embeddedness in a cohesive neighborhood are indeed positively correlated (*r* = 0.05, *p* < 0.001). Married cohabitation (*r* = 0.21, *p* < 0.001) and having children (*r* = 0.08, *p* < 0.001) are also positively related to religious attendance. This suggests belonging to a religious community could indeed result in ‘spill-overs’ in the neighborhood and family-domain.

Examining Model 5, however, where embeddedness in a cohesive neighborhood and family ties are included, we can see that although the effect of attendance at religious services has reduced somewhat (from 0.05 to 0.04), the effect of attendance at religious services is still significant. This small decrease indicates that embeddedness in the neighborhood and family plays a modest role at best in explaining why participation in a religious congregation is associated with higher levels of life satisfaction (as suggested in *hypotheses 3a* and *3b*). Hence, an effect of religious belonging exists that is independent of these secular ‘spill-overs’. This indicates that belonging to a religious community might also have a *cultural* social advantage, next to *structural* advantages in the form of embeddedness in non-religious social structures. However, embeddedness in the neighborhood and the family are not the only structural benefits of attendance at religious services: interactions and ties among churchgoers themselves might also lead to more social support and less loneliness. In other words, there might be structural benefits directly resulting from attendance at religious services. Therefore, we examine the role of social support and loneliness.

Inspecting the correlation of support and loneliness to attendance at religious services, we see that the relationship of attendance at religious services to social support is only moderate (*r* = 0.04, *p* < 0.01) and its relationship to loneliness is not significant (*r* = − 0.01, *p* = 0.34). To examine whether their role in mediating the association between attendance and life satisfaction is also negligible, we add the structural indicators social support and loneliness in Model 6, the full model. Turning to the effect of attendance at religious services, we see that the coefficient slightly decreases after adding social support and loneliness (from 0.04 to 0.03). For a small part, the role that attendance of religious services plays in the association between being Catholic and life satisfaction appears to be related to the provision of social support and the mitigation of loneliness, as put forward in *hypotheses 3c* and *3d*.

Importantly, however, attendance at religious services still plays a significant role when the influences of all the structural social benefits included in this study are taken into account. This residual effect of attendance at religious services is in accordance with what is proposed in the theoretical section: in addition to structural social advantages (more social ties and support, and alleviating loneliness), being Catholic also appears to be related to a cultural aspect of religious belonging, which entails sharing a common system of values and meaning in a religious community. This suggests *hypothesis 2* can be corroborated, which states that belonging to a community with a shared culture is an explanation for the positive effect of religious affiliation on life satisfaction.

We will discuss the implications of our results in the concluding section below.

## Discussion and Conclusions

Using the Netherlands as a strategic case to examine the association of religion with life satisfaction in a secularized, pluralistic context, this study shows a mixed picture. First, in contrast to previous findings on Christian affiliations in the United States, the understudied group of Muslims proves to be less instead of more satisfied with their lives than those who are non-religious. This result does, however, seem to be driven by their underprivileged social position, rather than any intra-religious causes. Second, we found no association between being Protestant and life satisfaction, while, third, being Catholic is associated with significantly higher levels of life satisfaction compared to those who do not have any religious affiliation. In order to explain why being Catholic is related to life satisfaction, we considered dimensions of religious believing and belonging.

Our findings indicate that it is crucial to discern different dimensions of religion that might be relevant to life satisfaction: not all dimensions of religiosity are the same in this respect, confirming that it is vital to uncover precisely *how* religion contributes to well-being (George et al. 2002). The conclusion that there is no ‘universal’ religion-effect is well-illustrated by our finding that while aspects of religious belonging play a significant role in explaining why Catholics are generally more satisfied with their lives, religious beliefs and private religious practices do not.

Existing literature suggesting that religiosity may have both negative and positive consequences for (mental) well-being could help explain this finding. Pargament et al. (1998) for instance introduced the concepts of ‘positive’ and ‘negative’ religious coping. An example of negative coping would be punitive religious appraisal, which is based on the idea that one has committed a sin for which one is being punished by God. This would explain why believing in God does not necessarily have a positive influence on one’s mental well-being. Since praying is also linked to different coping styles (Nooney and Woodrum 2002), conceptions of negative religious coping could also help explain why praying is negatively related to well-being in this study. In addition, because praying is often used to deal with problems, it may lead individuals to focus more on these problems, which could harm their mental health (Masters and Spielmans 2007). To identify how religious beliefs and praying play a role, future research could provide a more in-depth examination of the potentially cross-pressuring positive and negative relationships between various types of religious belief and prayer and well-being. This could also help to further explain why differences exist in how effective different religions are in enhancing, maintaining, or recovering mental well-being.

In contrast to religious beliefs, belonging plays an important role for the higher levels of life satisfaction among Catholics. Our findings suggest that there are two aspects of religious belonging that are relevant: a structural aspect based on the benefits derived from social ties, and a cultural aspect based on the idea that communities foster a sense of belonging through a shared culture or value system. More specifically, our finding of a residual effect of attendance at religious services when neighborhood embeddedness, family ties, support, and loneliness are taken into account suggests that cultural aspects of participating in a congregation play a role. This is in line with psychological literature that identifies belonging as a basic human need and a source of motivation (see, e.g., Choenarom et al. 2005), and ties it to health outcomes (Hagerty et al. 1996). Future research could, however, benefit from specific measurements of belonging, allowing a more careful examination of the influence of religious belonging in general, and more specifically of the role that a shared culture plays in such a sense of community. In order to examine whether they offer similar benefits to life satisfaction, a comparison could also be made between binding worldviews that are considered to be religious in nature and ‘secular’ worldviews (e.g., nationalism).

Our results are based on analyses conducted with a relatively young sample (14–49 years old). Including older respondents could result in somewhat different findings. It is, however, likely that the importance of belonging is even greater among an older population. Because social engagement and feelings of loneliness are even more important to the elderly (Golden et al. 2008), the social benefits of religion (both structural and cultural) could prove to be especially valuable to them. Although future research in this field should include a broader age range to be certain, our findings probably provide a moderate estimation of the role of belonging in the relationship between religion and life satisfaction.

As this study has been conducted with recent Dutch data, it is important to note that a sense of belonging provided by religious communities could be especially useful in a highly pluralistic context. As Kinnvall (2004) argues, the increased movement of ideas, people, and goods has brought about a sense of rootlessness, leading individuals to seek reaffirmation of their identity. Belonging to a religious community may be a particularly effective way of countering this contemporary sense of rootlessness (e.g. Kinnvall 2004; Seul 1999). The role of the national context in shaping how religion impacts life satisfaction should therefore be further examined in cross-national research.

## Footnotes

1 We included the following variables next to those already included in our regression analyses: respondent’s region, housing, living situation of the respondent during childhood, educational level of both parents, marital status of parents during childhood of the respondent, employment status of parents during childhood of the respondent, participation in highbrow cultural activities (e.g. visit the opera, museums, theatre, reading books) of both parents, religious affiliation of the father, inclination towards social desirability, locus of control, attitudes on upbringing of children, a verbal ability test, a numerical ability test, the respondent’s plans for the future, and an item indicating whether the respondent had any comments on the survey after finishing it.
